# Sensory modulation of gait characteristics in human locomotion: A neuromusculoskeletal modeling study

**DOI:** 10.1371/journal.pcbi.1008594

**Published:** 2021-05-19

**Authors:** Andrea Di Russo, Dimitar Stanev, Stéphane Armand, Auke Ijspeert

**Affiliations:** 1 Biorobotics Laboratory, École polytechnique fédérale de Lausanne, School of Engineering, Institute of Bioengineering, Lausanne, Switzerland; 2 Kinesiology Laboratory, Geneva University Hospitals and University of Geneva, Geneva, Switzerland; Northeastern University, UNITED STATES

## Abstract

The central nervous system of humans and other animals modulates spinal cord activity to achieve several locomotion behaviors. Previous neuromechanical models investigated the modulation of human gait changing selected parameters belonging to CPGs (Central Pattern Generators) feedforward oscillatory structures or to feedback reflex circuits. CPG-based models could replicate slow and fast walking by changing only the oscillation’s properties. On the other hand, reflex-based models could achieve different behaviors through optimizations of large dimensional parameter spaces. However, they could not effectively identify individual key reflex parameters responsible for gait characteristics’ modulation. This study investigates which reflex parameters modulate the gait characteristics through neuromechanical simulations. A recently developed reflex-based model is used to perform optimizations with different target behaviors on speed, step length, and step duration to analyze the correlation between reflex parameters and their influence on these gait characteristics. We identified nine key parameters that may affect the target speed ranging from slow to fast walking (0.48 and 1.71 m/s) as well as a large range of step lengths (0.43 and 0.88 m) and step duration (0.51, 0.98 s). The findings show that specific reflexes during stance significantly affect step length regulation, mainly given by positive force feedback of the ankle plantarflexors’ group. On the other hand, stretch reflexes active during swing of iliopsoas and gluteus maximus regulate all the gait characteristics under analysis. Additionally, the results show that the hamstrings’ group’s stretch reflex during the landing phase is responsible for modulating the step length and step duration. Additional validation studies in simulations demonstrated that the modulation of identified reflexes is sufficient to regulate the investigated gait characteristics. Thus, this study provides an overview of possible reflexes involved in modulating speed, step length, and step duration of human gaits.

## Author summary

This study investigates the possible reflex parameters that the central nervous system could use to modulate human locomotion. Specifically, we target the modulation of three gait characteristics: speed, step length, and step duration. We utilize human locomotion simulations with a previously developed reflex-based model and perform multiple optimizations ranging targeting low to high values of the three gait characteristics investigated. From the data acquired in optimizations, we investigate which reflex parameter correlates most with the gait characteristics changes. We identified nine key reflex parameters affecting gait modulation, performed validation experiments, and verified that the optimization of key reflex parameters alone could generate modulation in the studied locomotion behaviors. Kinematics, ground reaction forces, and muscle activity obtained in simulations show similarities with past experimental studies on gait modulation. Therefore, the identified parameters could potentially be used by the nervous system to regulate locomotion behaviors in a task-dependent manner. Other circuits not modeled in this study could play a crucial role in gait modulation, and further investigations should be done in the co-optimization of feedforward and feedback circuits.

## Introduction

The interactions between the nervous system and the musculoskeletal system allow humans and other animals to move and interact in their environment, choosing among different motor patterns through a complex and redundant interaction of neural circuits. However, the strategies used to control the different gait patterns have not been elucidated yet. It is well-known that the central nervous system controls locomotion in a hierarchical and distributed way by modulating the activity of its control subsystems such as spinal reflexes and central pattern generators (CPGs) [1], [2]. These networks are modulated by descending cortical and brainstem pathways and sensory feedback to regulate the motor outputs for the required motion [3–7]. It is also well-established that the modulation of sensory feedback relies on the control of reflex responses through reciprocal and presynaptic inhibition [8]–[10]. In mammals and lower vertebrates, stereotyped movements are executed with low sensorimotor gains, whereas locomotion relies on higher feedback gains during demanding tasks or unfamiliar conditions [9]. These observations led to the conclusion that the central nervous system modulates sensory transmission by adjusting reflex gains to develop adaptive responses to the environment. In addition to gains, specific reflexes such as the stretch response can also be modulated through changes of their activation threshold [10]. These reflex’s features are cyclically regulated in stereotyped rhythmic activities like locomotion [8] and are subject to task-dependent modulation [10]. Furthermore, feedback pathways might be more important than central circuits in controlling human biped locomotion. In fact, experiments on subjects with lost limb proprioception demonstrated that the amount of motor control delegated to sensory feedback is more prominent in humans compared to other mammals and lower vertebrates [11–15]. However, it is not yet fully understood which of the mentioned reflex’s features are tuned nor at what time during the gait cycle in order to modulate human locomotion.

Neuromusculoskeletal simulations are powerful tools to test hypotheses in neuroscience. In particular, past studies used these tools to investigate the interaction between biomechanical properties, sensory inputs, and spinal circuits [16–18]. Several proposed models aimed to reproduce the healthy behavior of human locomotion and its modulation. Many studies explored the modulation of feedforward CPGs circuits able to generate changes in gait speed [19–26], demonstrating how the modulation of selected feedforward components in the spinal cord can reproduce a wide range of walking behaviors. However, these models do not consider the potential effect of reflex circuits’ modulation in changing the gait characteristics. On the other hand, other studies highlighted the contribution of sensory feedback to motor control. Ogihara and Yamazaki gave one of the first contributions in this direction with a neural controller composed of motoneurons receiving inputs from a common CPG and reflexes from stretch and force receptors [27]. Additionally, in that model, the spindle reflexes included inhibitory inputs to antagonist muscles, and the parameters were optimized using genetic algorithm optimization. Subsequently, Geyer and Herr developed a purely reflex-based neuromechanical model reproducing kinematics, dynamics, and muscle activation of human walking behavior without the contribution of any CPG circuit [28]. The modulation of speed for this model was explored by Song and Geyer [29]. Performing different optimizations for six different speeds ranging from 0.8 to 1.8 m/s, they identified nine key reflex control parameters that show a significant trend and increase speed. Three key parameters were related to trunk balance, three to stance behavior, and three to swing generation. The model could generate speed transitions from slow to fast speed, optimizing the identified parameters. However, these key parameters did not include only reflex mechanisms but also balance, prevention of overextension, and reciprocal inhibition mechanisms that were simplified in a way that could hardly be related to specific physiological proprioceptive information. Subsequently, the same authors added supraspinal layers on top of a generalized reflex model in the three-dimensional space [30] able to reproduce walking and running behaviors [31]. These behaviors were achieved by optimizing stance reflex parameters and the modulation of two supraspinal parameters: desired foot placements and the desired minimum swing leg length. Therefore, gait modulation relied on integrating these descending pathways without identifying and selecting the feedback circuits contributing to this modulation. Moreover, some components of the control mechanisms were still difficult to translate in physiological meaning. More recently, Ong et al. developed a detailed reflex-based controller modeling each control component based on the physiology of proprioception with mechanisms for trunk balance and gait phase switching being the only non-physiological components [32]. The model is designed to walk in sagittal plane, and it is optimized for different target speeds between 0.50 m/s and 2.00 m/s reproducing kinematic, kinetic, and metabolic trends observed in human walking experiments [33]. This present study will use the model proposed by Ong et al. in order to identify the reflexes taking part in the modulation of speed, step length, and step duration.

The aforementioned studies demonstrated that both feedback and feedforward controllers could faithfully reproduce various walking behaviors highlighting the complexity of the neural and musculoskeletal systems as highly redundant mechanisms [34], [35]. CPG-based models partially uncovered the contribution of spinal feedforward oscillatory mechanisms generating diverse walking behaviors with CPGs parameters’ modulation. However, previous reflex-based models could not identify physiologically relevant reflex parameters responsible for gait modulation. Furthermore, previous studies in neuromechanical simulations focused mainly on achieving different target speeds rather than separate the components controlling step length and step duration. Lim et al. investigated the relations between these gait characteristics in an experimental study where subjects walked with combinations of small, nominal, and large step lengths (i.e., 0.584, 0.730, and 0.876 m) and short, nominal, and long step durations (i.e., 0.43, 0.52, and 0.65 s) [36]. The resulting range of speeds was between 0.89 and 2.04 m/s. The study showed that the activity of gluteus maximus, gluteus medius, vastus, gastrocnemius, and soleus are dedicated to vertical support and forward progression independent of changes made to either step length or step duration. In addition, increased step length results majorly from the larger contribution of hip and knee extensors.

This study aims to understand the potential mechanisms of task-dependent reflex modulation behind various behaviors of human locomotion. In particular, we aim at answering the following questions:

1. To what extent can the modulation of reflexes modify speed, step length, and step duration during walking?
2. Can these quantities be controlled independently?
3. Which specific reflexes should be modulated to adjust each quantity?

It is undoubtedly true that other mechanisms such as modulation of CPGs, muscle synergies, vestibular, visual, and cerebellar circuits also play an essential role in modulating locomotion. However, the present goal is to focus on the potential role of reflex modulation in gait adaptation and to analyze to what extent reflex modulations alone could explain the modulation of some gait characteristics. More specifically, we aim to understand the effect of the task-dependent modulation of the reflex circuits rather than the neural mechanism that performs this modulation since this would require the modeling of much more complex supraspinal neural networks. Therefore, in the implemented neuromechanical simulations, the spinal feedback mechanisms are isolated from human walking’s other neural components. Other neural circuits essential for locomotion, such as the vestibular feedback and the cyclic activation of spinal reflexes, are simplified to engineered components like a state machine and proportional derivative controls. The primary focus of this study is dedicated to the modulation of speed together with the independent modulation of step length and step duration. Three different sets of optimizations having various target speeds, step lengths, and step durations are performed. The results suggest that the reflex-based model can generate different gait behaviors, including low and high speed, step length, and step duration. Furthermore, walking patterns ranging among small and large step lengths could be achieved by maintaining the step duration fixed and vice versa. Finally, all these behaviors can be controlled with the modulation of nine identified reflex parameters that showed the highest correlation with the changing of gait characteristics.

## Materials and methods

The analysis performed in this study is conducted using the optimization and control framework SCONE [37]. The musculoskeletal model and the reflex controller are based on the ones used by Ong et al. [32] and are described in more detail in the following sections. We present the musculoskeletal model, the reflex controller, the optimization protocol, the description of the dataset analysis, and the validation steps.

### Musculoskeletal model

**Fig 1:**
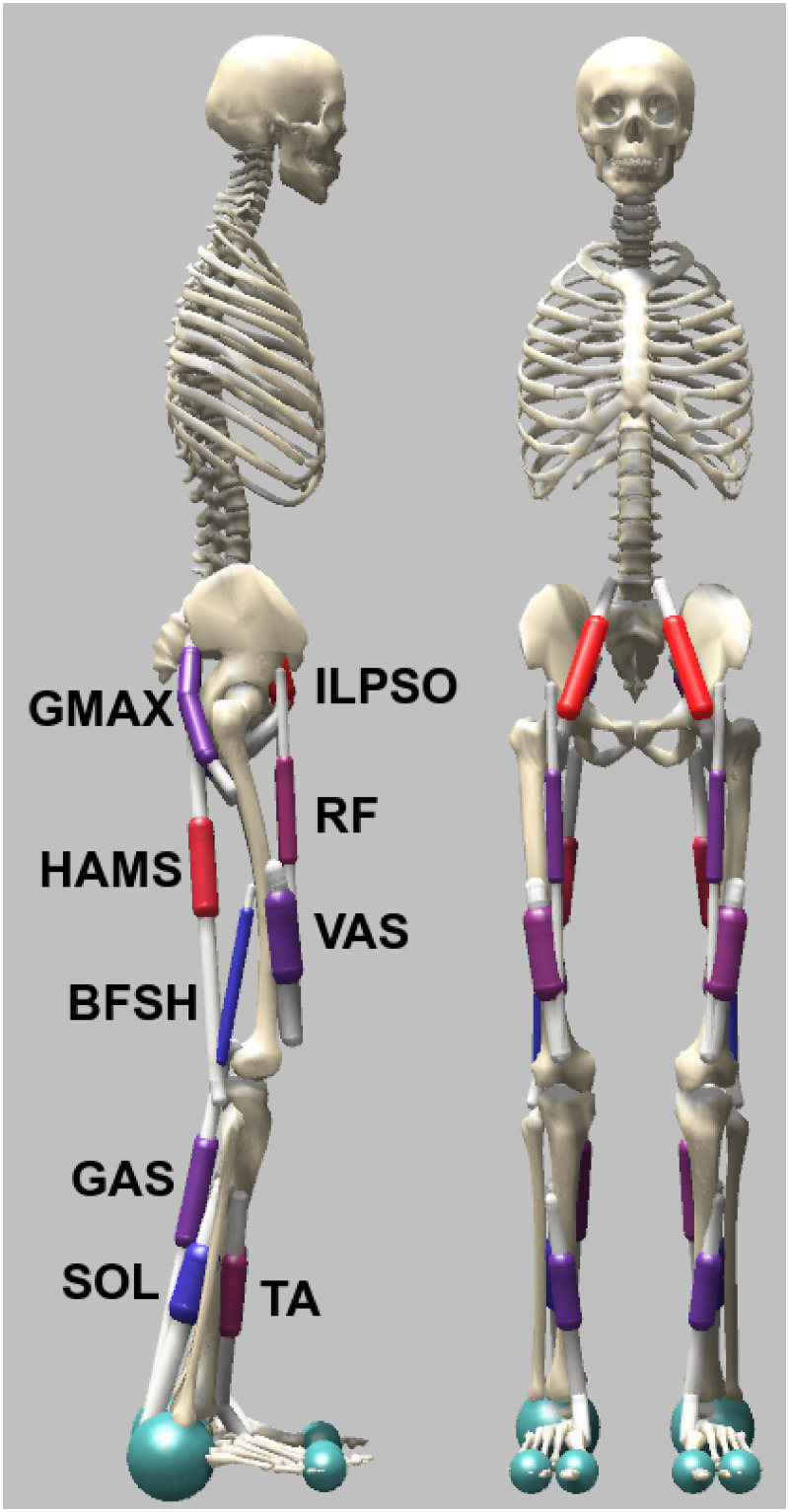
Musculoskeletal model used to study human locomotion. The model is constrained in the sagittal plane and has 9 DoFs: hip and knee flexion/extension, ankle plantar/dorsal flexion for each leg, and a 3-DoFs planar joint between the pelvis and the ground. The movements are generated by the activation of 9 muscles per leg: gluteus maximus (GMAX), biarticular hamstrings (HAMS), iliopsoas (ILPSO), rectus femoris (RF), vasti (VAS), biceps femoris short head (BFSH), gastrocnemius medialis (GAS), soleus (SOL), and tibialis anterior (TA).

The musculoskeletal model (Fig 1) is based on the one developed by Delp et al. [38], which is composed of a skeleton of height = 1.8 m and weight = 75.16 kg. The model movement is constrained in the sagittal plane and has nine DoFs in total: a 3-DoFs planar joint between the pelvis and the ground and other 3 for each leg, one at the hip, one at the knee, and one at the ankle. Three spheres are also included as contact model to estimate the ground reaction forces when they touch the ground. The contact model is taken from [39] and is composed of one bigger sphere of radius equal to 5 cm at the anatomical reference of calcaneus and two smaller of radius 2.5 cm at the anatomical reference of toes. The model is also composed of nine Hill-type muscle-tendon units [40] per leg: gluteus maximus (GMAX), biarticular hamstrings (HAMS), iliopsoas (ILPSO), rectus femoris (RF), vasti (VAS), biceps femoris short head (BFSH), gastrocnemius (GAS), soleus (SOL), and tibialis anterior (TA).

### Reflex controller

In the reflex controller proposed by Ong et al. [32] that we reused in the present study, the type of stimulation provided to each muscle depends on the phases of the gait cycle. The gait cycle is divided into five different gait subphases, three for the stance phase and two for the swing: early stance (ES), mid-stance (MS), pre-swing (PS), swing (S), and landing preparation (LP). Taking as reference the division of the gait cycle defined in clinical gait analysis [41], it is possible to classify the division in subphases proposed by Ong et al. [32] as follows:

- early stance (ES): first double support and early stance in single support
- mid-stance (MS): mid and late stance in single support
- pre-swing (PS): second double support
- swing (S): early and middle swing
- landing preparation (LP): late swing

The controller is based on three different kinds of feedback: positive force feedback from the Golgi tendon organs, length and velocity feedbacks based on the muscle spindles’ stretch reflex. Furthermore, Proportional Derivative (PD) controllers regulating the trunk’s forward-lean angle are integrated in the hip muscles’ stimulation to help maintain balance. A constant feedforward stimulation only dependent on the state of the gait cycle is also integrated. The types of stimulation provided to muscles are mathematically described in the equations below:

Feedforward stimulation:

$$\begin{array}{c} u_{C}=k_{C} \end{array}$$ (1)

Length feedback:

$$\begin{array}{c} u_{L}=k_{L}\cdot max\left(0,\left(\tilde{l}\left(t-t_{D}\right)-l_{0}\right)\right) \end{array}$$ (2)

Velocity feedback:

$$\begin{array}{c} u_{V}=k_{V}\cdot max\left(0,\tilde{v}\left(t-t_{D}\right)\right) \end{array}$$ (3)

Force feedback:

$$\begin{array}{c} u_{f}=k_{f}\cdot \tilde{f}\left(t-t_{D}\right) \end{array}$$ (4)

PD balance controller:

$$\begin{array}{c} u_{PD}=k_{p}\left(\theta \left(t-t_{D}\right)-\theta_{0}\right)+k_{v}\dot{\theta }\left(t-t_{D}\right) \end{array}$$ (5)

where *k*_*C*_ is a constant, *k*_*L*_, *k*_*V*_ and *k*_*F*_ are the gains of the reflex controller, *l*_0_ is the length offset of the stretch response. This offset defines the threshold value of the muscle length after which the length feedback produces a stimulation to the muscle itself. Concerning the stimulation given by the PD balance controller, *k*_*p*_ and *k*_*v*_ are the proportional and derivative controller’s gains, and *θ*_0_ is the desired forward lean angle regulating the proportional feedback of the actual forward lean angle *θ*. On the other hand, *t*_*D*_ represents the parameter for the time delay and it depends on the muscle proximity to the vertebral column: *t*_*D*_ = 5*ms* for the hip, *t*_*D*_ = 10*ms* for the knee, and *t*_*D*_ = 20*ms* for the ankle. The variables used in the controller (muscle length *l*, contraction velocity *v* and force generated *f*) are taken normalized according to specific muscle parameters: optimal length (*l*_*opt*_) and maximum isometric force (*f*_*max*_).

$$\begin{array}{c} \tilde{l}=\frac{l}{l_{opt}},\;\tilde{v}=\frac{v}{l_{opt}},\;\tilde{f}=\frac{f}{f_{max}} \end{array}$$ (6)

Finally, a state controller regulating threshold parameters that define the switching between one sub-phase of gait and another is also integrated. In this context, muscle activation is linked to the stimulation given by the controller through first-order dynamics:

$$\begin{array}{c} \frac{da}{dt}=\frac{u-a}{\tau } \end{array}$$ (7)

where *a* represents the muscle activation, *u* the muscle stimulation, and *τ* the dynamic time constant equal to 0.01 s. Furthermore, from the equations above, the stimulation provided to muscle depends on the muscle state (force, length or forward-leaning of the trunk) and independent parameters represented by the constant feedforward stimulation *k*_*C*_, the reflex gains *k*_*L*_, *k*_*V*_ and *k*_*F*_, the length offset of the stretch reflex *l*_0_ and the parameters of the PD controller *k*_*p*_, *k*_*v*_ and *θ*_0_. These parameters are the ones optimized to evaluate the ability of reflexes to modulate human gait. For this reason, the parameters under study are only the reflex gains and offsets since the other feedback mechanisms such as the PD balance controller are necessary to stabilize the gait but do not rely on physiological evidence.

In the next sections of this study, the reflex parameters are indicated with the following notation:

$$\begin{array}{c} KX_{muscle}^{subphases}\;\text{for}\;\text{the}\;\text{reflex}\;\text{gains}\\ L0_{muscle}^{subphases}\;\text{for}\;\text{the}\;\text{length}\;\text{offsets} \end{array}$$

where *X* represents the type of feedback (either *L* (length), *V* (velocity) or *F* (force)) *subphases* specify in which subphase of the gait cycle the reflex referred by the parameter is active (either ES, MS, PS, S, LP), and *muscle* represents the specific muscle targeted by the reflex (either ILPSO, GMAX, RF, HAMS, VAS, BFSH, GAS, SOL or TA). The parameters presented in the Results section are also included in the list of abbreviations.

### Optimization

The Covariance Matrix Adaptation Evolutionary Strategy (CMA-ES) method is used to optimize the parameters and obtain the different walking behaviors. The optimization parameters are the maximum number of generations equal to 1500, the samples per iteration λ = 16, and the step size *σ* = 1.

The cost function used is divided in seven different components:

- A penalty preventing the falling condition: the termination height parameter is used to detect if the model has fallen. This parameter is defined as the ratio between the center of mass (COM) height to the initial state. The simulation is terminated in advance when $\frac{COM\_height}{initial\_COM\_height}<th=0.8$ (termination-height). The amount of this penalty is defined by Eq 8.
  $$\begin{array}{c} p_{stability}=w_{stability}\cdot \left(\frac{time_{max}-time_{sim}}{time_{max}}\right) \end{array}$$ (8)
  where *time*_*max*_ represents the maximum simulation’s duration set to 15 seconds, *time*_*sim*_ is the time after which the simulation is terminated (*time*_*max*_ = *time*_*sim*_ if the model does not fall), and *w*_*stability*_ is the weight assigned.
- A penalty minimizing the difference between the target speed and the model’s actual speed:
  $$\begin{array}{c} p_{speed}=w_{speed}\cdot |speed_{model}-speed_{target}| \end{array}$$ (9)
  where *speed*_*model*_ represents the actual average speed recorded in the simulation, *speed*_*target*_ represents the desired speed to optimize, and *w*_*speed*_ the weight assigned.
- A penalty minimizing the difference between the target step length and the model’s actual step length:
  $$\begin{array}{c} p_{sl}=w_{sl}\cdot |sl_{model}-sl_{target}| \end{array}$$ (10)
  where *sl*_*model*_ is the average step length recorded in simulation, *sl*_*target*_ is the desired step length and *w*_*sl*_ is the assigned weight.
- A penalty minimizing the difference between the target step duration and the model’s actual step duration:
  $$\begin{array}{c} p_{sd}=w_{sd}\cdot |sd_{model}-sd_{target}| \end{array}$$ (11)
  where *sd*_*model*_ is the average step duration recorded in simulation, *sd*_*target*_ is the desired step duration, and *w*_*sd*_ is the assigned weight.
- A penalty minimizing the walking effort: the effort is computed taking the implementation of Umberger metabolic model [42], [43] with updates from Uchida [44]:
  $$\begin{array}{c} p_{effort}=w_{effort}\cdot effort \end{array}$$ (12)
  Computing the total heat rate (*tot*_*heat*_*rate*), the mechanical work rate (*mech*_*work*_*rate*), and the mass for each muscle, the effort is obtained according to Eq 13
  $$\begin{array}{c} effort=\frac{basal\_energy+\sum_{n=1}^{N_{muslces}}\left(tot\_heat\_rate_{i}+mech\_work\_rate_{i}\right)\cdot mass_{i}}{distance\cdot mass_{model}} \end{array}$$ (13)
  According to Uchida’s model, the basal energy is computed as 1.2 ⋅ *mass*_*model*_.
- A penalty minimizing the overcoming of joints ranges: this penalty is linked to the distance between the actual joints angle values and the desired joints ranges. Desired joints ranges in degrees are defined as [-16.2, 42.0] for the hip (positive flexion), [-1.3, 68.2] for the knee (positive flexion), and [-27.7, 15.5] for the ankle (positive dorsiflexion) [33]:
  $$\begin{array}{c} p_{joints}=w_{joints}\cdot |joints\_ranges_{model}-joints\_ranges_{target}| \end{array}$$ (14)
  where *joints*_*ranges*_*model*_ represents the joints ranges recorded in simulation, *joint*_*ranges*_*target*_ represents the desired joints ranges, and *w*_*joints*_ is the assigned weight.
- A penalty minimizing the head’s acceleration for head stability: the acceptable ranges in *m*/*s*^2^ where no penalty is applied are [-4.9, 4.9] for the vertical direction and [-2.45, 2.45] for the forward direction.
  $$\begin{array}{c} p_{head}=w_{head}\cdot |head\_acc_{model}-head\_acc_{target}| \end{array}$$ (15)
  where *head*_*acc*_*model*_ represents the head acceleration recorded in simulation, *head*_*acc*_*target*_ represents the desired ranges for head acceleration, and *w*_*head*_ is the assigned weight.

The final value of the cost function is given by Eq 16:

$$\begin{array}{c} cost\_function=p_{stability}+p_{speed}+p_{sl}+p_{sd}+p_{effort}+p_{joints}+p_{head} \end{array}$$ (16)

Three different sets of optimizations are performed with different targets implemented in the objective functions:

- Optimization set 1: different target speeds ranging from slow to fast gait
- Optimization set 2: different target step lengths ranging from small to large maintaining a fixed value of step duration
- Optimization set 3: different target step duration ranging from small to large maintaining a fixed value of step length

The first set of optimizations changes the target speed in every optimization covering a wide range, from the slowest to the fastest speed that the model’s stability can handle. The following optimizations for step length and step duration are performed starting from the initial condition of the best solution found in the mid-range at 1.0 m/s of speed with a value of step length and step duration around 0.7 m and 0.7 s, respectively. The second set investigates the modulation of step length having this gait characteristic as target varying in the different optimizations and a fixed target step duration in the range of [0.68, 0.72] s. Similarly, for the third set, the step length is kept fixed to the defined range of [0.68, 0.72] m, and the step duration is the varying target. Different weights are assigned to each cost function’s component depending on the optimization set. These weights are shown in Table 1.

**Table 1:** Assigned weight for each component of the cost function for the three optimization sets.

	set 1	set 2	set 3
*w*_*stability*_	100	100	100
*w*_*speed*_	100	100	100
*w*_*sl*_	0	1	0
*w*_*sd*_	0	0	1
*w*_*effort*_	1	0.1	0.1
*w*_*joints*_	0.1	0.1	0.1
*w*_*head*_	0.25	0.25	0.25

Since forcing a fixed step length or step duration while varying the gait characteristics increases the task’s effort, the weight for effort minimization is reduced from 1 to 0.1 for the last two sets of optimizations, not to penalize the task’s achievement.

### Solutions’ selection

The three sets of optimizations obtained contain several solutions where the achieved values of speed, step length, and step duration are recorded together with the amount of effort and joint ranges. Among these solutions, the ones selected for the current study satisfy the following conditions:

- stability: from the solutions that reached the maximum simulation time without falling condition, the stable solutions are the ones that show a convergence toward a constant oscillation of joint angles.
- effort: the efficient solutions from the energy point of view are the ones with an effort value lower than 4 *J*/(*kg* ⋅ *m*) for speed between 0.8 and 1.3 m/s, and lower than 8 *J*/(*kg* ⋅ *m*) for slower and faster speeds since these types of gaits requires higher energy expenditure.
- joints limits: the solutions selected for the analysis are the ones that have a penalty lower than 0.5 for the knee and hip angles. In contrast, the ankle angle’s penalty limit is set to 3 since the model tends to have high dorsiflexion.

Additionally, the second and third set of optimization also need to maintain the values of step duration and step length in the ranges reported previously. From the data obtained, it is possible to evaluate the three gait characteristics’ ranges when the human model’s movement is driven by reflexes allowing to answer the first research question.

### Dataset analysis

For the identification of key parameters, the focus is purely on reflex circuits. Therefore, the parameters related to balance, feedforward stimulation, and thresholds are not analyzed. The identification of critical parameters for gait modulation is found by examining the correlation coefficient between the parameter and the variation of the gait characteristics analyzed. Therefore, a reflex parameter is considered a candidate key parameter for gait modulation if it presents a correlation coefficient larger than 0.6 for at least one of the three gait characteristics’ modulation. Then, each identified parameter is analyzed through three different regressions, one for the solutions obtained by each of the three optimization sets. The regression analysis is performed with a similar methodology as previously done by Van der Noot et al. [26]. However, we take into consideration all the good solutions extracted instead of their average. Then, data are regressed, finding the lowest order polynomial function that can model the distribution with a coefficient of determination (*R*^2^) larger than 0.7. In case the solutions extracted are widely spread, the maximum polynomial order allowed is set to three.

### Validation of gait behaviors

The previous stage allowed us to identify the parameters that mostly correlate with gait modulation. However, in the case of stretch reflexes, the same stimulation level can be achieved by modulating the gain (*k*_*L*_) or the length offset (*l*_0_). Therefore, when one of these stretch parameters is identified as a key parameter, the other belonging to the same reflex is also considered a key modulator for the validation study. Once the key reflexes are identified, we demonstrate that the variation of these is sufficient to achieve the same ranges of gait variability achieved in the previous experiments. This process is done by performing new optimizations exploring the gait characteristics’ boundaries (minimum and maximum speed, step length, and step duration). During these optimizations, reflexes that were not identified as relevant are not allowed to change, and their value is kept constant. On the other hand, the key reflexes are optimized together with the parameters regulating balance, feedforward stimulation, and states. Further validation is done optimizing only the non-relevant reflexes for the gait modulation and keeping the key parameters to a constant value with the target objective of obtaining the same boundaries of the gait characteristics obtained previously. This process is done to demonstrate the model’s reduced ability to modulate the gait without the possibility to change the key reflex parameters. The other parameters not belonging to the reflex controller are also optimized in this case. Other experiments explored the capability of key parameters to modulate the gait characteristics without the contribution of PD balance controller and states’ threshold since also these non-physiological parameters may contribute to gait modulation.

The identification of key reflexes and the validation process permit to answer to the last two research questions allowing to define which reflex controls which gait characteristics and if these characteristics can be controlled independently. The study then presents the gait analysis of joints kinematics, ground reaction forces, and muscular activity, considering minimum, intermediate, and maximum values of the obtained speed, step length, and step duration.

## Results

This section presents first the gait limits that the model can reach in terms of speed, step length, and step duration, then the identified key parameters. These parameters are divided depending on whether they control step length, step duration, or both. The identification of the key parameters is made by analyzing the linear correlation for the three gait characteristics and the tendency of the data distribution shown together with the regression model.

Three different sets of optimizations were performed. Specifically, set 1 contains 147 solutions extracted from 12 optimizations, whereas set 2 and set 3 respectively include 134 and 75 solutions, both extracted from 9 optimizations from each set. Table 2 shows the minimum and maximum boundaries reached during the optimization processes. From the first one investigating the speed modulation, the solutions were extracted within the range of speed from 0.45 to 1.71 m/s, step length from 0.45 and 0.87 m, and step duration from 0.51 to 1.04 s. The solutions in the second set of optimizations achieved a minimum step length of 0.45 and a maximum of 0.88 m, covering the same range already obtained in the first set with an increasing target speed. The step duration was maintained constant at 0.69 s, and this condition has been satisfied for all the solutions selected with a tolerance of 0.01 s. Consequently, the range of speed obtained in this second set is reduced compared to the first one because of the imposed fixed step duration, which is included between 0.69 and 1.48 m/s. Finally, the third set presents solutions with values of step duration ranging from 0.51 to 0.91 s. The target step length is maintained fixed to 0.72 m, and the solutions extracted satisfied this condition with a tolerance of 0.02 m. The range of speed obtained out of this optimization set is included between 0.78 and 1.46 m/s.

**Table 2:** Boundaries of the three gait characteristics targeted during the three optimization sets. The first set shows the optimizations’ results where the gait target to reach is the desired speed that varied from the lower to the upper boundary. The second set shows the optimizations’ results with a fixed step duration and a varying target step length. Finally, the third set shows the optimizations’ results with a fixed value of step length, changing the target step duration.

Optimization set [min, max]	Speed [m/s]	Step length [m]	Step duration [s]
Set 1	[0.45, 1.71]	[0.45, 0.87]	[0.51, 1.04]
Set 2	[0.69, 1.48]	[0.45, 0.88]	[0.68, 0.70]
Set 3	[0.78, 1.46]	[0.70, 0.74]	[0.51, 0.91]

The changing of parameters’ values with gait characteristics’ modulation permits identifying the reflexes controlling speed, step length, and step duration. Table 3 presents the correlation coefficients of the nine key parameters identified. Among these, some reflexes are linked with specific gait characteristics. Specifically:

**Table 3:** Correlation coefficients of identified key reflex parameters: Three reflexes were found to have a significant effect on speed through the modulation of step length, four to have an impact on all the three gait characteristics, and two able to modulate step length and step duration accordingly with small effects on speed.

	Speed	Step length	Step duration
Step length modulators			
$L0_{HAMS}^{ES-MS}$	0.4892	0.7827	0.1312
$KF_{SOL}^{MS-PS}$	0.9082	0.8156	0.3632
$KF_{GAS}^{MS-PS}$	0.8978	0.7074	0.1348
Step length and step duration modulators with effects on speed			
$KL_{ILPSO}^{PS}$	0.6931	0.8029	0.6363
$L0_{ILPSO}^{S}$	0.4689	0.2328	0.7409
$KL_{GMAX}^{LP}$	0.7285	0.4041	0.6454
$L0_{TA}^{LP}$	0.8121	0.5978	0.3431
Step length and step duration modulators without effects on speed			
$KL_{HAMS}^{LP}$	0.1452	0.4781	0.6072
$L0_{HAMS}^{LP}$	0.3382	0.8707	0.7602

- three key reflexes for the modulation of speed by modulating only the step length.
- four key reflexes for the modulation of speed by modulating both step length and step duration.
- Two additional reflexes modulating both step length and step duration, such that together this results in keep speed more or less constant.
- no reflex parameter has been found to modulate step duration independently from step length.

Since we did not find parameters that could modulate step duration without significantly affecting step length, step duration is mainly modulated by reflexes that interact with step length’s regulation as well. Therefore, the solutions presented where step duration’s modulation is achieved maintaining the step length fixed could be obtained only with other reflex circuits’ compensation mechanisms.

### Step length modulators

**Fig 2:**
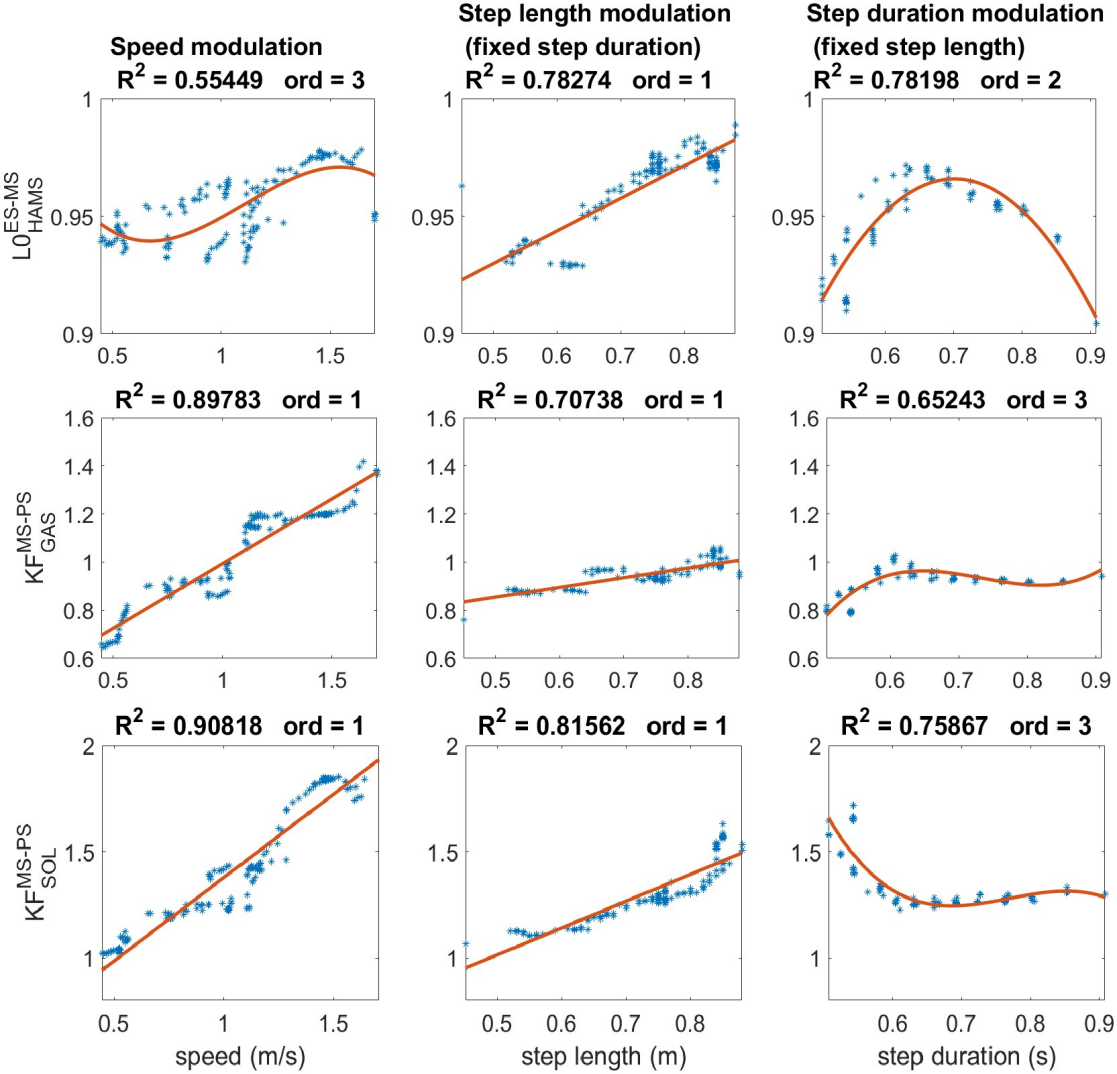
Regression analysis of step length modulators. The solutions obtained by the three sets of optimizations are represented by the blue dots, whereas the red curve represents the regression. The plot on the left shows the data distribution and regression for the speed modulation, while the plots on the center and the right show the step length and step duration modulation, respectively. The reflexes presented facilitate speed and step length increasing with minimal effect on the step period. The offset length of the hamstrings’ stretch reflex presents an influence on step duration modulation, but the global increasing tendency of speed reflects the behavior found in the modulation of step length.

In total, three parameters have been selected for step length modulation, namely $L0_{HAMS}^{ES-MS}$: (length offset of hamstrings’ stretch reflex during early and mid-stance), $KF_{GAS}^{MS-PS}$ (positive force feedback’s gain of gastrocnemius muscle during mid-stance and pre-swing), and $KF_{SOL}^{MS-PS}$ (positive force feedback’s gain of soleus muscle during mid-stance and pre-swing). These are the ones that showed a high correlation coefficient with speed and step length and a low correlation coefficient with step duration, indicating a minor effect on this latter gait characteristic. All these parameters regulate the stimulation given by reflexes activated during the stance phase. The length offset of the hamstrings’ stretch reflex in the first two sub-phases of stance shows a high correlation coefficient with the modulation of step length (*c* = 0.7827) and a lower correlation with the modulation of speed (*c* = 0.4892) and with a significant low correlation with the modulation of step duration (*c* = 0.1312). The graphs in the first row in Fig 2 show the linear relationship with the step length modulation (*R*^2^ = 0.7827). However, parameter variation also affects the step duration, as shown by the quadratic regression (*R*^2^ = 0.78198). Therefore, the same parameter value can generate two different gaits patterns with different step durations depending on the other reflex parameters’ compensatory mechanisms. This nonlinear effect on the step duration results in a less clear dependency between the hamstrings stretch offset and the increase of speed. In fact, the values of this parameter are very spread around the linear regression for the speed modulation. However, the global increasing tendency seems to follow the one found for step length modulation.

On the other hand, the propulsive muscles seem to have a key role in modulating step length. Indeed, both gastrocnemius and soleus’ positive force feedback has a high linear correlation with step length modulation with *R*^2^ = 0.81562 for soleus and *R*^2^ = 0.70738 for gastrocnemius. This linear dependency is also present in the speed’s modulation that can be modeled as a linear regression with *R*^2^ = 0.90818 for soleus and *R*^2^ = 0.89783 for gastrocnemius. The changing of these two parameters shows minor effects on the modulation of step duration with low correlation coefficients. Indeed, the parameters’ values were maintained roughly constant for longer step durations than 0.6 s. Nevertheless, shorter durations more proper of high speeds may affect the parameters’ values. Indeed, the positive force feedback gain of soleus increases when step duration goes below 0.6 s while gastrocnemius’ one decreases. It is also possible to notice that the value that the parameters can reach is limited if constraints to the gait characteristics are applied. This is especially true for the gastrocnemius’s positive force feedback gain that cannot reach values larger than 1.1 if a fixed step length or step duration is imposed.

### Step length and step duration modulators

The following results present the key parameters affecting both step length and step duration significantly. These parameters are separated into two groups: those that affect speed modulation and those that do not affect speed. This diversification is made because possible parameters that influence step length and duration coherently maintain the ratio between these two gait characteristics roughly constant, minimizing the effect on speed modulation.

#### With effects on speed

**Fig 3:**
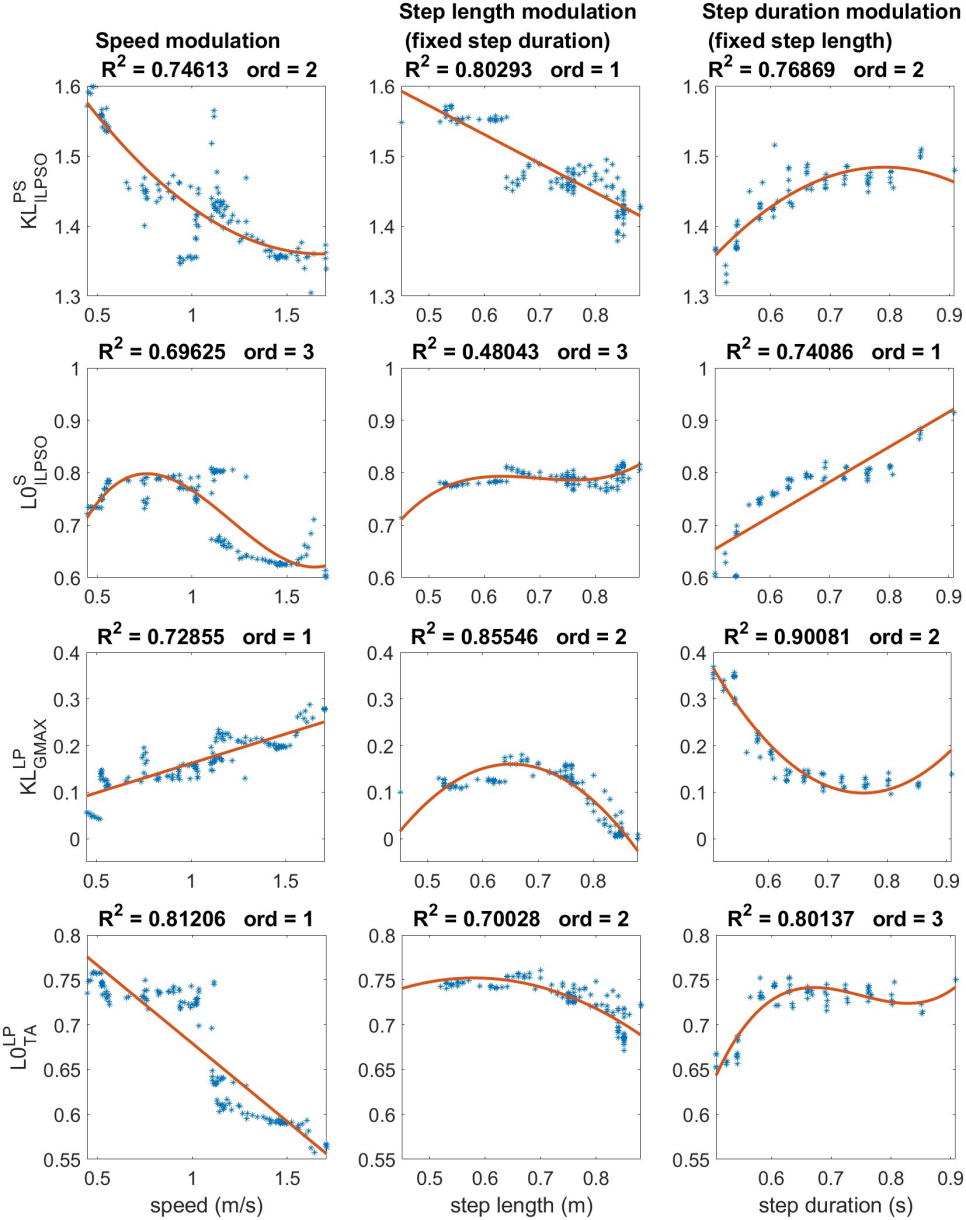
Regression analysis of step length and step duration modulators with effects on speed. The stretch reflex gain of iliopsoas during pre-swing and the length offset during swing have a decreasing impact on speed due to decreasing step length and increasing step duration for the former and primarily for step duration increasing for the latter. On the other hand, the stretch reflex gain of gluteus maximus in landing preparation has an increasing linear effect on the speed with nonlinear effects on step length and step duration. The length offset of tibialis anterior’s stretch reflex can modulate fast speed with large step lengths and short step durations but has less effect in the modulation of slow gaits.

$KL_{ILPSO}^{PS}$ (stretch reflex’s gain of iliopsoas muscle during pre-swing), $L0_{ILPSO}^{S}$ (length offset of iliopsoas’ stretch reflex during swing), $KL_{GMAX}^{LP}$ (stretch reflex’s gain of gluteus maximus muscle during the landing phase) and $L0_{TA}^{LP}$ (length offset of tibialis anterior’s stretch reflex during the landing phase) are the key reflex parameters that influence both step length and step duration, significantly affecting speed. The graphs at the top of Fig 3 described the behavior of the stretch reflex gain of the iliopsoas during pre-swing. This parameter decreases linearly with the increasing step length and increases with the rising step duration, resulting in a global decrease of speed.

In the following phase of the gait cycle, we can observe a decreasing length offset of iliopsoas’ stretch reflex during swing with the increase of speed. However, the parameter’s values start to decrease only after 1 m/s while it remains roughly constant for slower gaits, as described in the plots presented in the second row of Fig 3. The decrease with increasing speed is coherent with the step duration observed tendency that increases linearly with the increase of length offset value. The step length modulation shows that the parameter maintains values close to 0.8 except for small step lengths below 0.6 m, where the parameter slightly increases from 0.7 to 0.8. This dependency found for small step length contrasts with the step duration’s increasing behavior, possibly explaining the low effect of this parameter for slow speed modulation.

The length feedback gain of the gluteus maximus during landing linearly influences speed modulation. However, this linearity is not found in the modulation of step length and step duration as observed in the third row of Fig 3. The critical contribution on the modulation of step length is given mainly at values larger than 0.7 m showing a drastic decreasing behavior of the parameter. It is also possible to observe that the reflex gain increases from a value of 0.1 to 0.2 for small step lengths. This increasing behavior is probably the main contributor to the growing speed modulation behavior for slow speed. However, the parameter values keep increasing for the fastest speed, although they decrease for large step lengths. Therefore, at the fastest speeds, the modulation is given by the effect on the step duration that decreases rapidly when the reflex gain increases.

The last parameter for speed modulation is represented by the length offset of the tibialis anterior’s stretch reflex. Concerning speed modulation, despite the data being regressed efficiently with a linear function, the parameter values were kept at around 0.75 for slow speeds until reaching 1 m/s. After this speed value, the length offset constantly decreases and maintains a decreasing behavior for the fastest gaits. Coherent decreasing of the parameter’s value can also be found for large step length and short step durations typical of fast speeds. On the other hand, the parameter remains roughly constant for small step lengths and long step durations typical of slow speeds.

#### Without effects on speed

**Fig 4:**
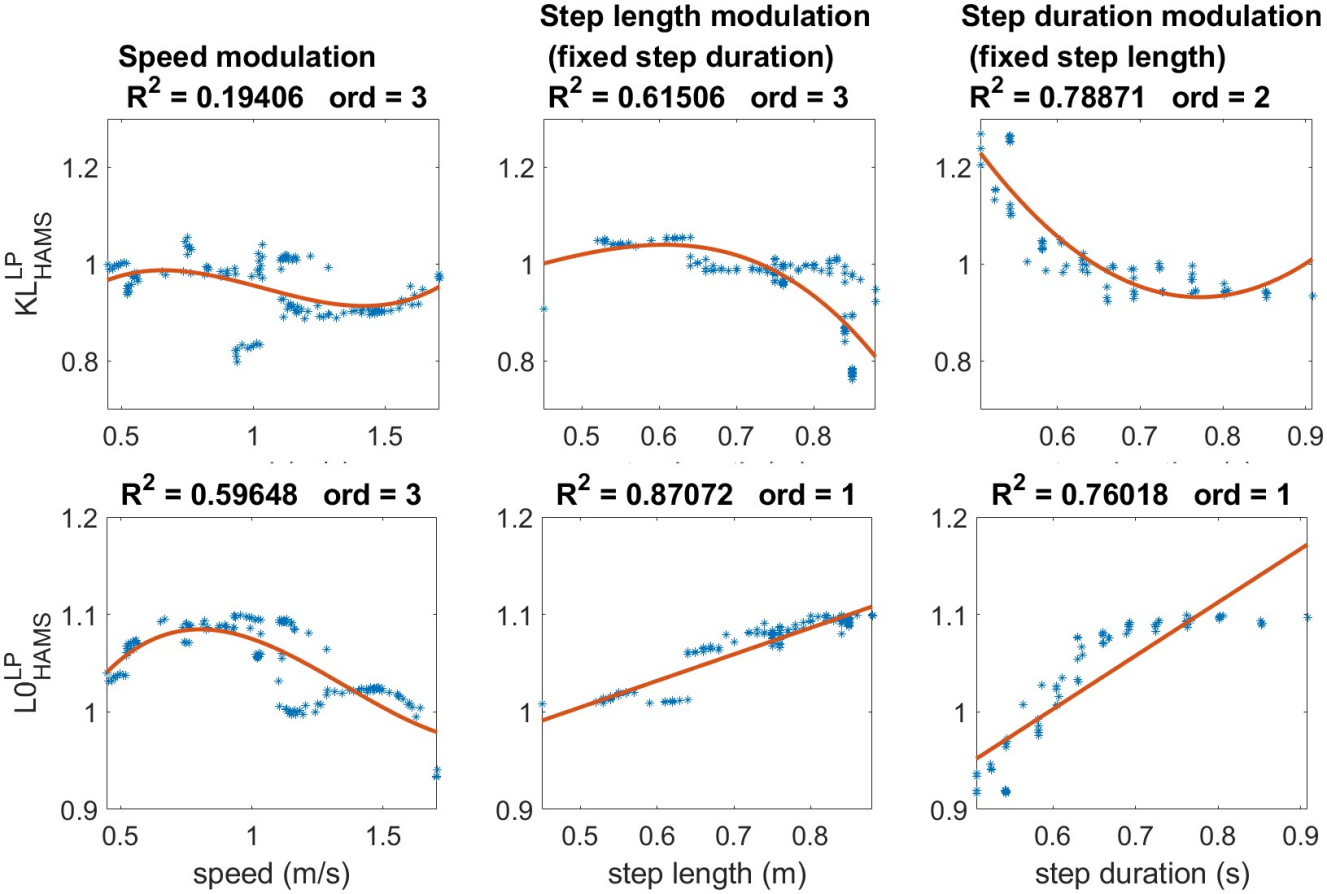
Regression analysis of step length and step duration modulators with effects on speed. Increasing the stretch reflex gain of hamstrings during landing preparation leads to a decreasing in step length and step duration resulting in a low influence in speed modulation. Similarly, the increasing length offset of hamstrings’ stretch relfex during landing preparation results in an increased step length and step duration with a small effect on speed modulation.

$KL_{HAMS}^{LP}$ (stretch reflex’s gain of hamstrings muscle during the landing phase) and $L0_{HAMS}^{LP}$ (length offset of hamstrings’ stretch reflex during the landing phase) control the modulation on both step length and step duration, maintaining their relation roughly constant with a minimal effect on speed. Both these parameters are related to the hamstring’s stretch reflex activity during landing preparation and are represented by the gain and the length offset. From the graphs at the top of Fig 4, the gain decreases accordingly with both step length and step duration resulting in a null effect on the speed modulation as shown by the spread distribution of data and from the low coefficient of determination of the third-order polynomial (*R*^2^ = 0.19406). The increasing length offset values with the increasing step length and step duration also contribute to the stretch response’s reduced activity when the two gait characteristics increase, as shown in the bottom graphs in Fig 4. Also in this case, the global effect is less efficient in the modulation of gait velocity.

### Modulation of key parameters

**Fig 5:**
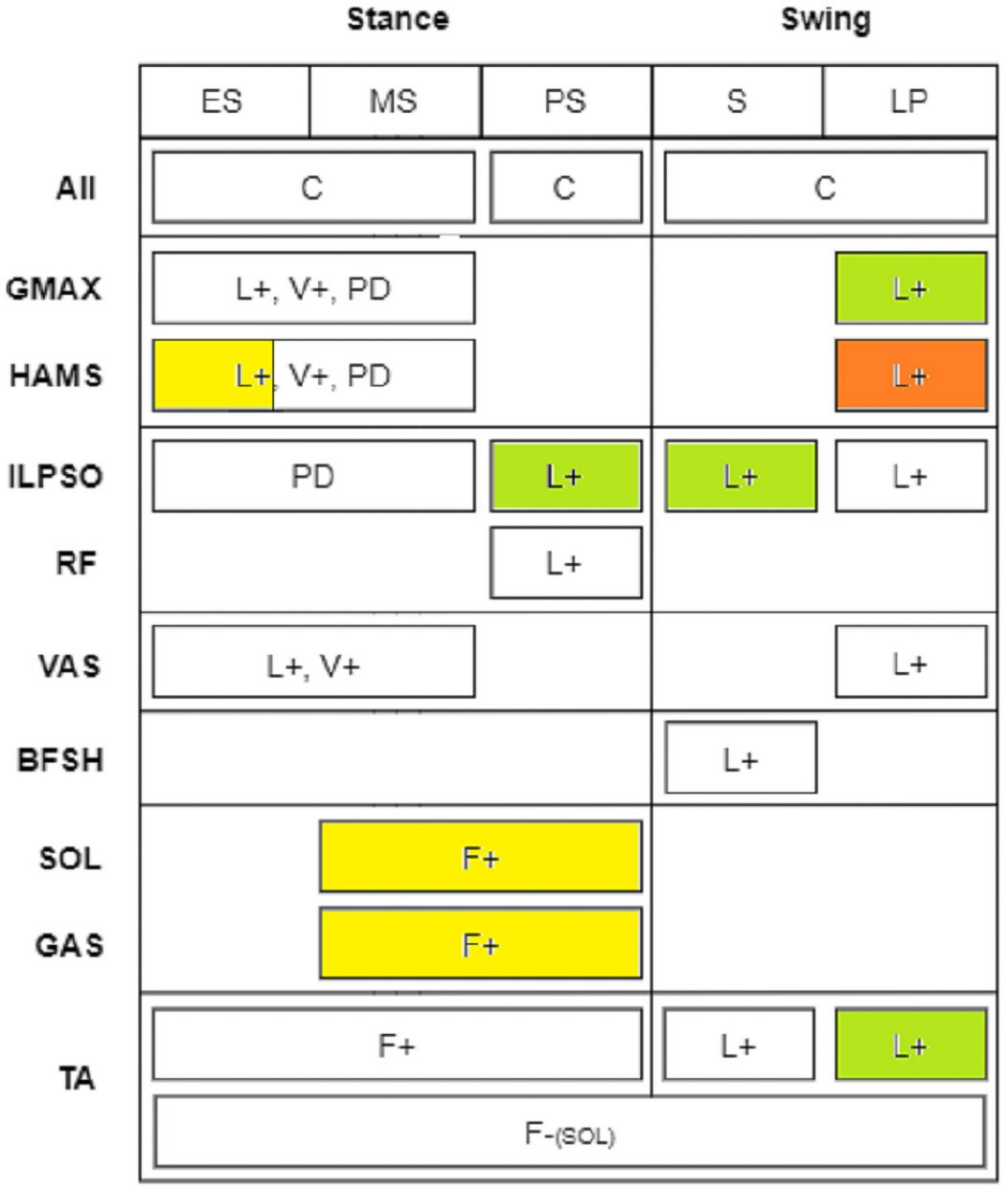
Diagram of the reflex controller with key reflexes modulating gait highlighted. The reflexes that were found to modulate mainly step length are highlighted in yellow. In contrast, those modulating step length and step duration together are highlighted in green and red depending on whether they showed a significant effect on speed (green) or not (red).

From the previous section, we were able to identify the reflexes that may affect the gait modulation. These key reflexes are highlighted in the control diagram of Fig 5. Step length modulators are highlighted in yellow, whereas step length and step duration modulators are highlighted in green and red depending on whether they have or do not affect speed, respectively.

This section presents the largest ranges reached for the modulation of the three gait characteristics, optimizing only the key reflexes identified together with the feedforward, balance, and state controller parameters. These boundaries are compared with the ones obtained previously, optimizing all the parameters. Another comparison is made with the achieved boundaries resulting from optimizations of all non-relevant parameters (not selected as key parameters).

Table 4 presents the largest boundaries obtained by optimizing the key reflexes identified. Comparing to Table 2, the modulation of key parameters could generate locomotion behaviors from slow to fast gaits with large and small step lengths and short and long step durations. The boundaries of the three gait characteristics cover the same ranges as those obtained with all reflex parameters’ optimization. Similar results are obtained for the modulation of step length and step duration. Therefore, the key reflexes selected demonstrated to be able to modulate gait with the same performances of the modulation of all reflexes.

**Table 4:** Boundaries of the three gait measures reached with the optimization of key reflexes. The optimization of the key reflexes alone could obtain the same performances of the gaits obtained optimizing all the reflexes suggesting that the major role of modulation is delegated to the key reflexes.

Optimization set [min, max]	Speed [m/s]	Step length [m]	Step duration [s]
Speed modulation	[0.48, 1.71]	[0.43, 0.88]	[0.51, 0.98]
Step length modulation	[0.77, 1.26]	[0.52, 0.87]	[0.69, 0.70]
Step duration modulation	[0.79, 1.30]	[0.70, 0.71]	[0.54, 0.91]

However, some reflexes that were not considered key modulators could still significantly affect locomotion modulation since the neural system is highly redundant. The results from the optimizations of non-relevant reflexes targeting the same ranges achieved in the previous stages show that the model fails to achieve slow and fast speed targets when the key identified reflexes are kept constant and not included in the optimization. For step length modulation maintaining a fixed value of step duration, the model cannot reproduce stable locomotion with a small step length. However, large step gaits could be achieved with similar performances obtained previously without modulating the identified reflex modulators. On the other hand, the optimization could not reach long step durations maintaining an intermediate value of 0.67 s when trying to target high values. However, the optimization could converge to a behavior able to reproduce short durations comparable to those previously obtained.

These results suggest that there are parameters beyond the key reflexes identified to modulate large step length and short step duration. These parameters do not necessarily belong to the reflex controller but could be part of the feedforward, balance, or state controller. In order to verify this, we performed optimizations varying the key reflex parameters alone and other optimizations varying only the other reflexes maintaining constant in all cases feedforward, balance, and state controller parameters. We verified that the modulation of state threshold parameters alone could achieve large step lengths. By fixing these parameters, we could reach a step length value of 0.87 m with the optimization of key parameters. In contrast, the optimization of other reflexes could only reach a step length below 0.8 m. On the other hand, the modulation of short step duration could be achieved with PD balance parameters’ contribution. Maintaining these parameters constant, a short step duration of 0.54 s could be achieved by optimizing the key reflex parameters. In comparison, the other reflexes could not converge to solutions with a step duration value lower than 0.62 s. Therefore, the key reflexes identified describe a large variance of the neural feedback mechanism’s modulation, whereas the other reflexes do not seem to affect gait modulation significantly.

### Gait analysis

**Fig 6:**
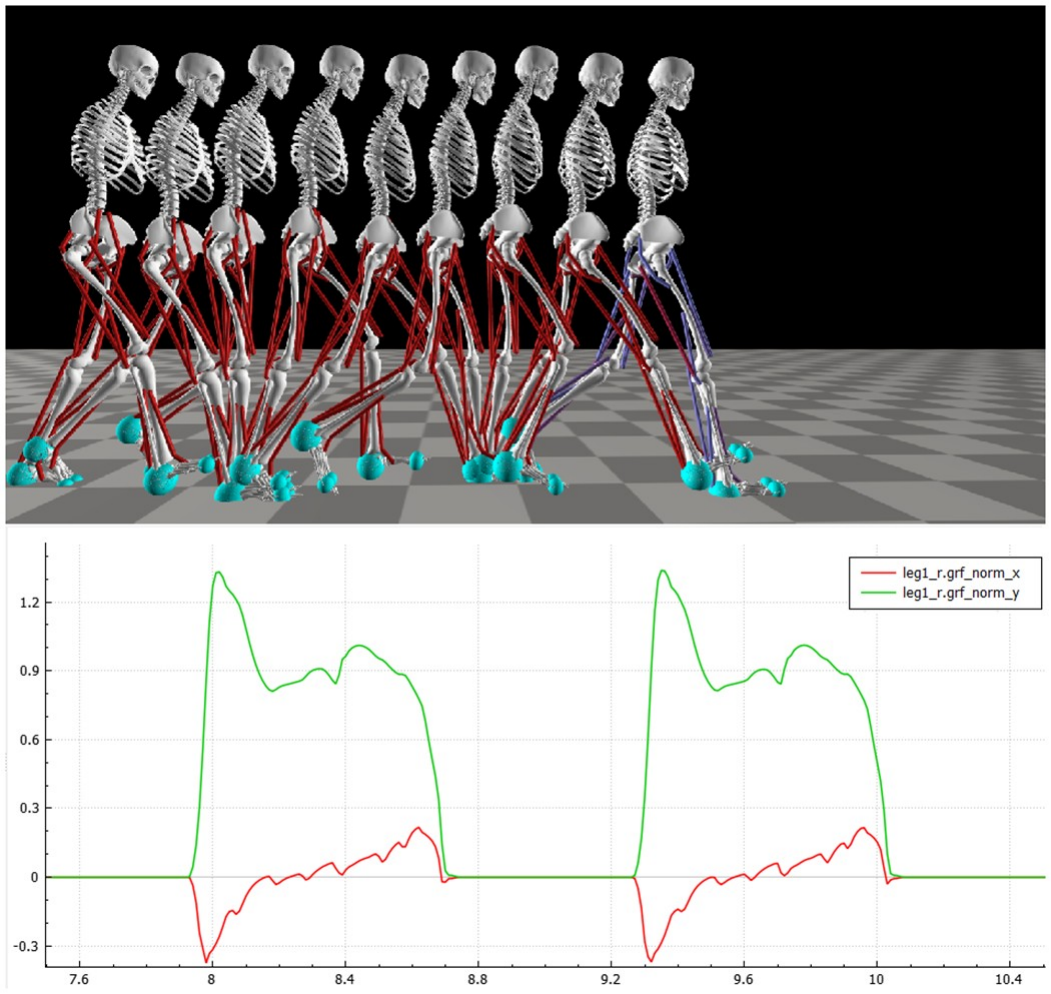
Representation of gait behavior with snapshots taken at different frames of for the speed modulation solution with target speed equal to 1.2 m/s. The instant taken is represented together with the behavior of vertical and horizontal ground reaction forces.

This section presents the gait analysis of the different solutions obtained, considering low, intermediate, and high values of speed, step length, and step duration when modulating the key reflexes. Firstly, the joints angles are presented, followed by ground reaction forces and muscle activation. These results are also compared to findings reported in past experimental studies [36], [45]–[47]. It can be firstly noted that the reflex controller could generate human-like locomotion behaviors as shown in Fig 6 for the specific solution from the first set of optimization at the intermediate speed of 1.2 m/s.

#### Kinematics and GRFs

**Fig 7:**
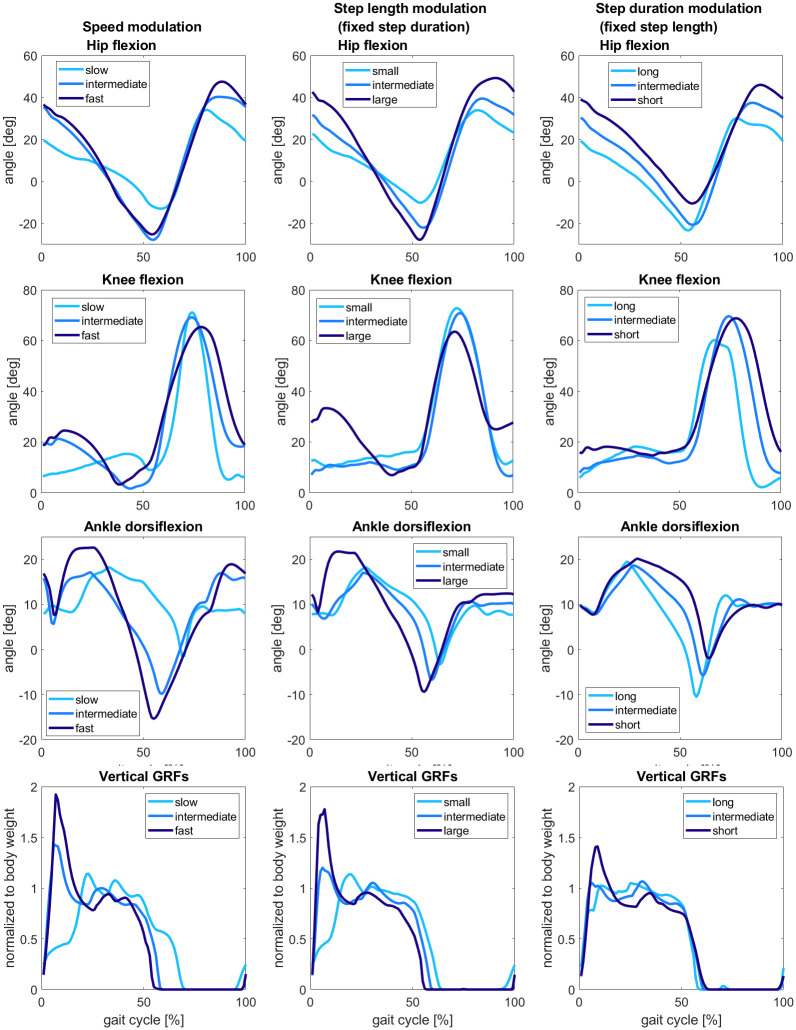
Joints angles and ground reaction forces for low, intermediate, and high values of the three gait characteristics. The cyan curves describe the slow gaits with small step lengths and long step durations, whereas the dark blue curves describe the fast gaits with large step lengths and short step durations. The change observed in speed modulation is closer to the only step length modulation than the modulation of step duration.

Fig 7 describes the behavior of joint kinematics and ground reaction forces. The hip angle has larger oscillation amplitudes for fastest speeds and largest step lengths. Wu et al., Schwartz et al., and Moissenet et al. also observed the same behavior in their experimental results about speed modulation [46], [33], [47]. Besides, Lim et al. observed that the hip flexion’s peaks were increasing with the step length’s increase, whereas no significant changes with step duration’s modulation are observed [36]. Indeed, our results show that, in these conditions, fast speed and large step lengths start from a flexion value around 40 degrees, decrease during stance to a maximum extension around -25 degrees and increase in swing to a maximum flexion value close to 50 degrees. By contrast, at slow speeds and small step lengths, the gait cycle starts with a hip flexion value of 20 degrees, decreases during stance to a minimum extension of -10 degrees, and increases during swing to a maximum extension value below 40 degrees. This behavior is not preserved in the modulation of step duration, where the hip flexion maintains roughly the same oscillation amplitudes with a tendency to maintain flexion for short step durations. Indeed, the hip flexion values vary from 30 to -20 degrees for long durations and from below 50 to -10 degrees for short durations. Therefore, the changing of speed shares more similarities with the changing of step length rather than step duration when considering hip flexion variation [36].

Then, for the knee flexion, we can observe that the modulation of speed step length and step duration does not significantly affect the knee flexion observed during the swing phase. Indeed, we can only observe that this flexion is around 70 degrees for all the proposed values of speed and step length with a small decrease of maximum flexion to 60 degrees for long step durations. However, it is possible to notice that difference is more important for the smaller knee flexion observed at the beginning of the stance phase. In fact, the amplitude of this flexion is larger with the increase of speed and can be observed only for large step lengths. Furthermore, the flexion is delayed to 40% of the gait cycle for very slow speed gait simulations. Concerning the modulation of step duration with fixed step length, the knee angle remains fully extended during the stance phase and starts to flex only at the beginning of the swing phase with the large knee flexion. These results slightly differ from the observations provided by experimental studies. Indeed, Wu et al., Schwartz et al., and Moissenet et al. observed a decrease in the small peak’s amplitude present in stance for slow speeds, as also observed in our experiments [46], [33], [47]. However, a decrease in the knee flexion in swing is reported for very slow speeds. In addition, Lim et al. noticed an increase in knee flexion for large step lengths and short step durations.

Concerning the ankle joint, compared to experimental results, there is excessive dorsiflexion between 10 and 20 degrees, and the plantarflexion is anticipated to 30% of the gait cycle instead of the 50%. The results show that the peak dorsiflexion in stance increases when speed rises. The same behavior can be observed for the peak plantarflexion in pre-swing. Furthermore, it is possible to notice a consistent delay of ankle dorsiflexion from 60 to 80% of the gait cycle for slow gait simulations. The curves’ tendency appears similar for speed and step length modulation. Concerning the effects of modulation of step duration, higher values for plantarflexion are reached with long durations, and it keeps on decreasing with the step duration’s decrease. The shown results seem to agree with the experiments performed by Wu et al. for the amplitudes of ankle dorsiflexion and plantarflexion [46]. However, it should be noted that Schwartz et al. and Moissenet et al. observed higher first dorsiflexion for very slow speeds compared to fast speeds [33], [47]. On the other hand, Lim’s experiments partially confirm our results since he observed that ankle plantarflexion increases with rising speed [36]. Nevertheless, he also observed that a decrease in step duration results in a slight increase in ankle plantarflexion, whereas in our results, this peak is kept small for short durations.

From the shapes of ground reaction forces, it is possible to observe a high peak at the beginning of the cycle for fast speeds, large step length, and short durations due to the fast collision with the ground. Furthermore, the second peak of the GRFs curve is anticipated compared to the ones usually found in experimental data. Moreover, the curve is flattened with small oscillations for slow speeds, small step lengths, and long step duration. These plots also provide information on the duration of the stance and swing phase in the gait cycle percentage. The stance phase ends around 60% of the gait cycle, except for slow gaits, where the stance phase is prolonged to 70%. These results resemble the observations found in experimental data from Wu et al. and Schwartz et al [33], [46].

#### Muscle activity

**Fig 8:**
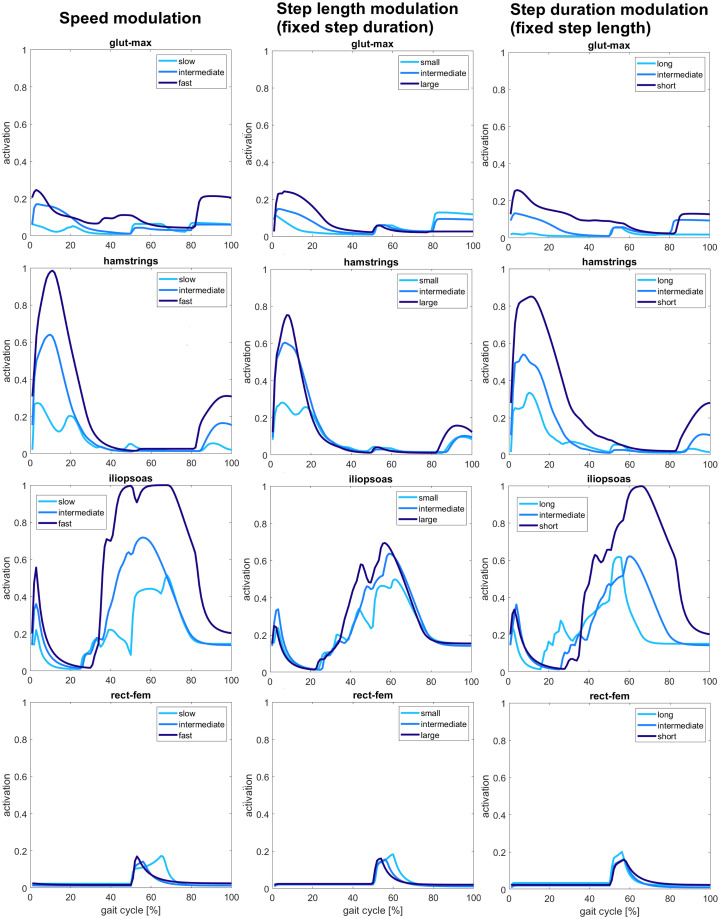
Hip muscles activity. Hamstrings and iliopsoas activity show a significant increase with increasing speed and step duration. Gluteus maximus activation is less evident, but it increases activation at the fastest speeds, whereas the rectus femoris does not show significant changes.

**Fig 9:**
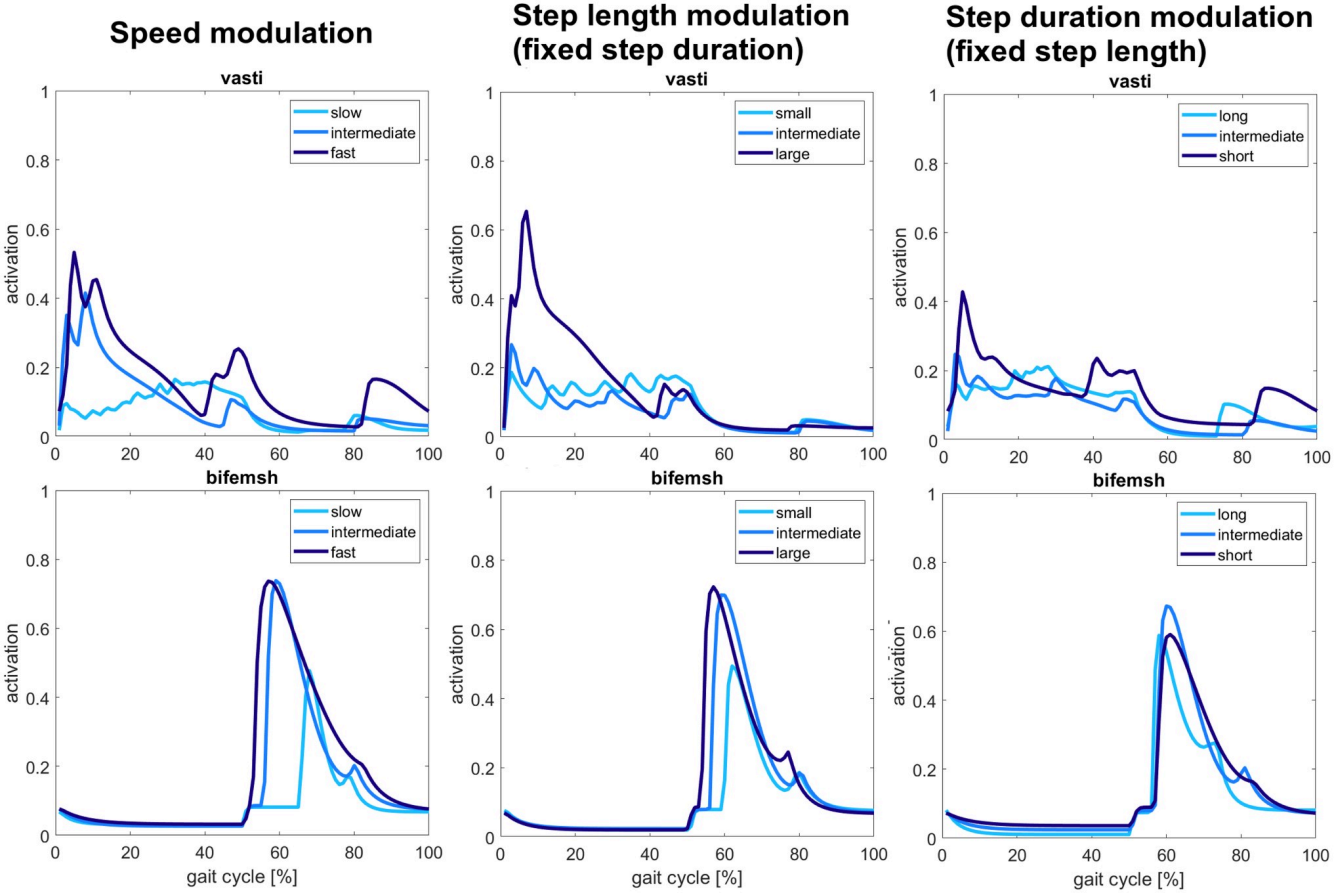
Knee muscles activity. Higher activation is observed for both the vasti and biceps femoris short head muscles at fast speeds and large step lengths.

**Fig 10:**
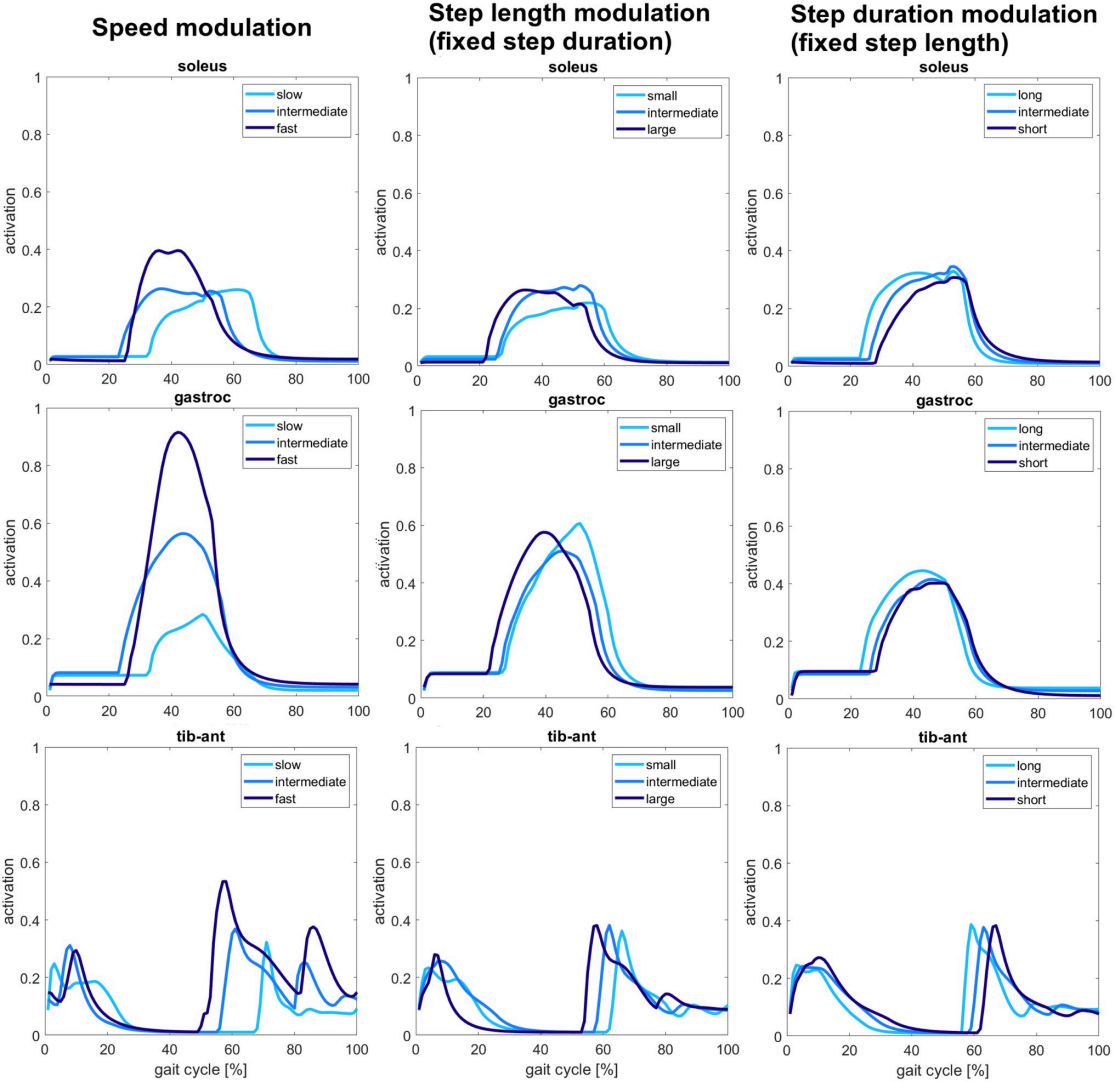
Ankle muscles activity. Soleus and gastrocnemius activity increase with the increase of speed, but no significant changes are seen for the modulation of step length and step duration. Similarly, tibialis anterior activity increases with speed, whereas for step length and step duration modulation, it can be observed anticipation and delay of activation during the gait cycle for large step lengths and short durations, respectively.

Figs 8, 9 and 10 show the muscle activity changes for the hip, knee, and ankle muscles, respectively. From the activation of the gluteus maximus, we noticed that the muscle is active mainly at the beginning and at the end of the gait cycle. It has a higher activity at fast speed during the stance phase despite the reflex gain and offset parameters have been maintained constant since it was not considered a key reflex for gait modulation. Higher activity at the beginning of the gait cycle is also observed at large step lengths and short step durations. Gluteus maximus activation reaches significant levels around 0.2 during the landing preparation due to the stretch reflex activity. This activation appears to be higher at fast speeds and short step durations but decreases significantly at large step lengths. Globally, the muscle never reaches a high activation through the gait cycle, as it is possible to see from the graphs where the activation is kept below 0.3. From the experimental studies performed by Ivanenko et al. and Lim et al., gluteus maximus’ peak activation increases with increasing speed and step length and decreases with increasing step duration, as also observed in our results [36], [45].

From the second row of Fig 8, the activation of hamstring muscle through the gait cycle in early stance and mid stance and during landing increases significantly with the increase of speed and step length and decreasing of step duration. In fact, hamstrings activation passes from values around 0.3 for slow speeds, small steps, and long durations to values around 0.8 for large step lengths and short durations until reaching peak values close to saturation for fast speeds. The single peak obtained is not preserved for slow speeds and small step lengths where the activation curves show a double peak behavior. During landing, the muscle activation also shows higher activity with increasing speed and decreasing step duration, passing from values around 0 to 0.3. A slight activity increase is also observed for gait with large step lengths with peak values below 0.2 and around 0.1 for small and intermediate step lengths. The experiments performed by Ivanenko et al., Schwartz et al., and Lim et al. also observed an increased activity with speed and step length’s increase and step duration’s decreases [33], [36], [45].

The iliopsoas muscle is active during early stance, pre-swing, and swing phases with decreasing activity during landing. The muscle activity during stance driven by the proportional derivative controller increases slightly with speed from 0.2 to 0.6, but no significant changes are present for step length and step duration modulation, maintaining the peak activation values around 0.3. The activity of iliopsoas during pre-swing and swing phases is significantly high for fast speeds and short duration, reaching the maximum activation level during these phases. Significant changes of increasing activity are also present between slow and intermediate speeds, with peak activation values rising from 0.4 to 0.7. Nevertheless, there is no significant difference in amplitude between long and intermediate durations for the step duration’s modulation where the peak activation values around 0.6. Still, the muscle activity decreasing during swing is anticipated for long durations. The increasing step length also increases muscle activation but less critically than the one observed for the speed modulation. In contrast, the peak activity ranges between above 0.4 and below 0.8 for step length modulation. On the other hand, Ivanenko et al. recorded a low EMG signal from iliopsoas for every speed value investigated [45]. However, Lim et al. found an increasing peak activity for large step length and short step durations, similarly to our results [36].

From the plots shown at the last row of Fig 8, the rectus femoris muscle is active only during pre-swing, and the activation does not change meaningfully for the different gait targets. The activation is kept low in all the conditions that do not overcome the level 0.2 of activation. These results do not match what observed in experiments from Ivanenko et al. and Schwartz et al., who recorded increasing activity with speed increase [33], [45].

From Fig 9 showing the knee muscles activity, the vasti muscle group is active mainly during early stance, mid-stance, and landing. The increasing of speed also increases muscle activation from 0.1 of slow speeds to 0.5 of fast speed. Other increases of activity are present for large step lengths during stance and short duration gaits during landing, raising the muscle activity from 0.2 to 0.6 and from 0.2 to 0.4, respectively. Lim et al. and Ivanenko et al. also observed an increased peak activation of vasti muscles, increasing speed, step length, and decreasing step duration [36], [45].

The activity of the biceps femoris short head is also higher with the increasing values of speed and step length. This increase is less consistent when passing from intermediate to fast speed than from slow to intermediate speed. Indeed, the peak activity increases from 0.4 for slow speeds and small step lengths to 0.7 for intermediate and fast speeds and intermediate and large step lengths. On the other hand, there is no consistent difference in muscle activity for the step period modulation that maintains the peak activity values to 0.6. No meaningful comparisons with experimental studies were possible since gait modulation experiments did not record this muscle activity.

From Fig 10, it is possible to notice an increased activity for soleus and gastrocnemius muscle during mid-stance and pre-swing when speed increases with the peak activation value passing from 0.25 to 0.4. These results are validated from experiments by Lim et al. and Ivanenko et al. [36], [45]. Furthermore, an activation delay is present for slow speeds. However, no other significant activity changes linked to the different gait targets are observed, with peak activation values kept to 0.3 for soleus and 0.5 for gastrocnemius. However, Lim et al. observed that both increasing step length and decreasing step duration resulted in increased soleus and gastrocnemius muscle activity. [36].

Finally, the tibialis anterior is active at the beginning of stance phase and during swing. Also in this case, the activity is increasing with increasing speed with a delay observed for slow gaits. In particular, peak activation values pass from 0.3 for slow speeds to 0.5 for fast speeds. A smaller delay is also observed for smaller step lengths and shorter step durations without a large amplitude variation. The experimental results from Ivanenko et al. and Schwartz et al. found similar results of our simulations for speed modulation [33], [45]. However, Lim et al. did not perform recorded the tibialis anterior muscle, and no comparisons with experimental studies are possible for step length and step duration modulation [36].

## Discussion

In this study, we aim first to understand how much a human model controlled by sensory-driven neural signals can replicate various gait behaviors at different speeds, step lengths, and step durations. Then, we aim to investigate whether these gait characteristics can be controlled independently, and finally to identify possible reflexes that the central nervous system can modulate in task-dependent locomotion. The results obtained from the optimizations show that the reflex controller could generate large ranges of these three gait characteristics. These results are coherent with the previous studies involving the application of sensory-driven controllers [28], [31], [32] that focused mainly on the single modulation of energy-efficient walking at different speeds. Our results demonstrated that sensory reflexes could modulate speed and energy-efficient gaits and control step length and step duration independently with less efficient gaits. These last generated behaviors can be performed by humans even though they are beyond their optimal energy efficiency. Furthermore, nine key reflex parameters are found to be good candidates to modulate human gait. Three of them modulate speed and step length, four modulate all the three gait characteristics, and two modulate step length and step duration accordingly with small effects on speed. These results demonstrate that the modulation of these key reflexes is sufficient to generate various human locomotion behaviors, ranging from reduced to high values of speed, step length, and step duration similar to those obtained with the optimization of all the reflexes. Therefore, the modulation of a small subset of reflexes could, in principle, be involved in the strategies used by descending commands to change the gait behavior together with the already investigated modulation of feedforward circuits ([21], [24]).

Some of the identified parameters, mainly active during stance, were found to influence speed and step length. Indeed, step length is affected by the level of propulsion that the stance leg muscles can give, pushing the body forward and lowering the center of mass. Coherently with experiments in human subjects [48], the soleus and gastrocnemius muscles give the main propulsion through their positive force feedback, as observed by the strong correlation that the two reflexes have with both speed and step length modulation. However, soleus and gastrocnemius muscle activation show a considerable change only with speed modulation. No significant variations are observed with the modulation of the step length. It should be observed that muscle activation does not depend solely on the values of reflex parameters. In fact, the stimulation given to the muscle depends on the reflex parameters and the muscle state. For this reason, the muscle activation that responds to stimulation as a first-order linear dynamics is also dependent on the state of the muscle itself. It has been verified that the positive force feedback gains of soleus and gastrocnemius change accordingly with the data distribution and regression laws described in the results section. Therefore, the unchanged activation level observed for the modulation of step length is due to an alteration of muscle states. An example can also be found in the activation of vasti that exhibits large variations depending on the target gait, although its reflexes are kept constant and are not modulated. This behavior is due to the state-dependent excitation provided to each muscle. Indeed, the muscle activation regulated by excitation may vary because of the different states that the muscle is having in different gait conditions (i.e., changing of the gait characteristics) even if the reflex parameters are kept constant. Therefore, the regulation that reflex parameters give to muscle excitation has the final effect of facilitating or preventing changes generated by the muscle state.

Another parameter affecting the step length is the length offset of the hamstrings’ stretch reflex during the stance phase. More precisely, increasing the length offset of the hamstrings’ stretch reflex results in a faster gait with larger steps. By contrast, decreasing this parameter results in a slower gait with small steps. Indeed, the length offset defines the muscle fiber length level after which the stretch reflex is active. Therefore, a larger length offset allows the hamstrings to sustain a stretch level due to the knee extension and hip flexion. This condition mainly happens in early stance at higher step lengths. Thus, an increased length offset prevents large stretch reflex responses that would produce an undesired knee flexion.

On the other hand, gait modulation with significant effects in all the gait characteristics relies largely on reflexes active during swing preparation or swing phases. The main stretch activity that plays a role in this modulation is the one governing iliopsoas’ activity. In fact, the larger length offset of this muscle causes a slower response of the stretch activity resulting in a slower and lower leg lifting typical of gait with reduced speed. Moreover, the decreasing stretch activity of iliopsoas during the swing preparation allows a large hip extension necessary for larger steps when the step length increases.

Then, the fast execution of landing is guaranteed with the modulation of gluteus maximus and hamstring stretch reflexes. The activity of the gluteus maximus during the landing phase is crucial to determine the step period since its higher stretch response allows a faster landing of the foot, increasing the frequency of the gait. On the other hand, the stretch reflex of hamstrings during the landing phase regulates the coherent increasing or decreasing of step length and step period through the regulation of both reflex gain and offset. The increased activity of hamstrings helps a faster landing phase due to the hip, but it also prevents a full extension of the knee, slightly reducing the step length. The excessive knee flexion is prevented by regulating the length offset that tends to increase linearly with step length. Nevertheless, this regulation tends to slow down the reflex’s stretch response less effective in fast landing.

The modulation of reflex parameters described above involves the regulation of sensory-motor gains (*k*_*F*_ and *k*_*L*_) and the threshold for the onset of the stretch response (*l*_0_). Physiologically, the modulation of gains can be obtained with the involvement of presynaptic inhibition of afferent activity [49], [50]. Besides, the regulation of descending modulation and *γ*-motoneurons is thought to contribute to the stretch reflex threshold’s modulation [51]. The altered regulation of this reflex component also takes a role in generating motor impairments in gait pathology [52].

For the comparison with experimental studies, we found a good match with kinematics and ground reaction forces, especially for hip angles showing higher flexion and extension peaks with increasing speed, step length. Concerning the knee flexion, experiments observed lower peaks in knee flexion in swing for very slow gaits. However, we do not find this behavior in our simulations. Still, it should be pointed out that our sensory feedback controller is not able to replicate very slow walking that in experiments reach values of 0.1 m/s, whereas our model cannot walk at speeds below 0.4 m/s. The ankle angle is the one that shows the most important limitations in our simulations. In fact, the model tends to have excessive dorsiflexion in all the solutions found, and in some of them, it shows anticipation in the plantarflexion movement. This behavior is probably also causing the anticipation in the second peak of vertical GRFs. However, the ankle angle and GRFs’ modulation resemble the behaviors observed in experiments with larger plantarflexions and higher peaks when speed increases.

Concerning the muscle activation, the results are consistent with the past experimental studies. The muscles that present the most relevant activity changes are the hamstrings, the iliopsoas, and the gastrocnemius. In simulations, the iliopsoas muscle is also active during the initial stance phase, whereas it is active only during pre-swing and swing in human experiments. This behavior is due to the PD controller that stimulates iliopsoas muscle in early stance to maintain the model’s balance. Furthermore, human experiments in speed modulation result in a low EMG signal for the iliopsoas muscle, while in our simulations, it appears to have high activity, especially for fast gaits. However, it should be noticed that experimental studies using surface EMGs are limited from the muscle deepness. Indeed the iliopsoas is located deep in the trunk, and it is not easy to record its activity [53]. Besides, Yokoyama et al. estimated the motoneurons’ activity in the spinal cord and found that the activation ratio between lumbar and sacral segments increases with speed [54]. Therefore, proximal muscles controlled by lumbar segments increase their activity more consistently than distal muscles controlled by sacral segments. We also observe this condition in our results. Indeed, there is a more significant increase in iliopsoas and hamstrings muscle activity than the increasing soleus and gastrocnemius activity when passing from slow to fast speeds.

Finally, the key reflexes’ variation achieved the same gait behaviors obtained by optimizing all the controller parameters from the validation performed. Furthermore, not allowing the key parameters to change resulted in a severe limitation of gait modulation. Physiologically, the neural system may modulate additional feedback circuits for human locomotion than the one identified. Yet, the results suggest that the identified reflexes are sufficient to modulate the gait in the contest of purely sensory-driven mechanisms.

Other experimental studies tried to obtain information about the neural circuitries involved in locomotion indirectly. Ivanenko et al. performed experiments applying vibration to hamstrings muscles [55]. These local effects of vibration can be explained in the light of a lengthening illusion of the vibrated muscle in that phase of the gait cycle where the muscle is lengthened [56]. The results showed that the perturbation to the hamstrings muscle evokes increments in walking speed. In our results, we similarly observed an influence of the hamstrings stretch reflex on walking speed in the first two subphases of stance. Nevertheless, Ivanenko et al. noticed that phasis stimulation was more effective in swing rather than in stance. On the other hand, Verschueren et al. applied tendon vibration separately to tibialis anterior and calf muscles [56]. The results showed a decreased plantarflexion at toe-off when the perturbation was applied to tibialis anterior. In contrast, a decreased dorsiflexion was observed when the same perturbation was applied to the calf muscles. In the end, the authors suggest the involvement of Ia afferent input in the online control of joint rotations. Our study includes stretch Ia excitation for tibialis anterior, but not for calf muscles (soleus and gastrocnemius). However, a progressive decline in soleus H-reflex excitability has also been observed from standing to walking and from walking to running due to an increased Ia presynaptic inhibition [57–64]. Besides, an increased stimulation of the tibialis anterior would probably result in excessive dorsiflexion weakening the plantarflexion in toe-off, as observed by Verschueren et al. [56]. Other studies investigated the effect of transspinal stimulation on short-latency tibialis anterior flexion reflex during walking [65]. The reflex facilitation occurred at heel contact and then progressively from late stance reaching its peak at early and late swing phases. These results are coherent with our model’s structure where the stretch reflex of the tibialis anterior is active in all the phases of the gait cycle, and it is strongly inhibited by the soleus muscle leading to a peak activity during the swing phase. However, it should be observed that experiments performed increasing the afferent activity stretching the muscle or by electrical stimulation reveal the effect of the added afferent activity on top of the already ongoing natural baseline activity. Therefore, these experiments investigate primarily the impact of a sudden external perturbation rather than the contribution of afferent activity in locomotion [66].

A more reliable approach could be the removal of afferent feedback through the effect of sudden unloading. These kinds of experiment mainly investigates group Ib fibers’ activity since these are more sensitive to load feedback than group Ia or II [67]. Klint et al. used a robotic platform changing the surface’s inclination to apply small dorsiflexion and plantarflexion perturbations to the ankle joint in early stance [68]. The results showed that in soleus and gastrocnemius muscles, the modulation of activity increased with inclines and decreased with declines suggesting these muscles to be modulated mainly through Ib fibers. This observation is coherent with the choice done in our controller to regulate plantarflexor activity with positive force feedback stimulation. In the context of task-dependent reflex modulation, significant studies were conducted applying acceleration and deceleration impulses delivered at the time of heel strike during treadmill walking [69]. It has been observed that the gastrocnemius muscle was inhibited during deceleration and excited during acceleration linking polysynaptic spinal pathways activating gastrocnemius with the gait speed. This behavior is also observed in our analysis where the positive force feedback gain of gastrocnemius muscle increases its amplitude according to the increasing walking speed.

Furthermore, Gerasimenko et al. gave a fascinating point of view in the context of sensory control in human locomotion [70]. They proposed that conceptually all sensory information in real time provided to the brain and spinal cord can be considered as a feedforward phenomenon. Indeed, the central nervous system processes sensory input in a feedforward manner to make fundamental decisions defining motor responses. Therefore, the sensory feedback system integrates into feedforward networks, and it is continuously regulated by them. In this context, the task and phase dependent spinal reflexes’ modulation explored in this study can be seen as part of these feedforward mechanisms in charge of regulating the gait characteristics.

Although this study’s results largely reflect findings reported in past experimental studies, some limitations need to be highlighted. First, the lack of DoFs above the pelvis delegates the balance control to hip muscles that are generally not involved in trunk balance. Indeed, the PD controller efficiently stabilizes the gait. However, this mechanism is only a first approximation of the complex neural networks dedicated to regulating balance in human walking involving vestibular and cerebellar systems. The modeling of these networks is very challenging and would require a significant increase in the number o parameters. Nevertheless, the detailed representation of balance mechanisms is beyond the scope of this study. Then, the non-convex nature of optimization and the optimizer’s stochastic nature lead to the conclusion that the found solutions are probably not the global minima. This difficulty is perhaps the reason why our results present excessive dorsiflexion and anticipation in ankle plantarflexion. Furthermore, the controller investigated could not achieve extremely slow speeds below 0.4 m/s that we can observe in human experiments [46]. This is probably because, at those speeds, there is a major involvement of descending control from brain areas (e.g. for balance control) that are not modeled in the reflex-based controller. Besides, the proposed controller relies on a state machine mechanism for the cyclic activation of reflexes, the control of which is most probably delegated to mutual inhibition of antagonist muscle or rhythmic circuits located in the spinal cord. Indeed, central pattern generators may play a crucial role in modulating locomotion through phasic feedforward signals and temporal activation of spinal circuits. In our study, we decided to focus on the modulation of sensory feedback mechanism alone since abstract models of central pattern generators basing their rhythmic patterns on sensory feedback and muscle synergies have already demonstrated to be able to modulate walking and running behaviors [21], [24], [25].

The neuromotor control of gait modulation is a highly redundant problem. Multiple combinations could have similar effects since the model’s simplification does not consider other circuits likely to influence gait modulation. Nevertheless, considering that direct experiments are challenging to perform on humans, this modeling approach could be informative and of significant value concerning the spinal sensory circuits that high-level centers may modulate to change gait characteristics. In this context, the modulation of key parameters investigated should be considered as one of the many possible strategies that the central nervous system may perform to modulate human gait. Future works should focus on the combined modulation of feedforward and feedback circuits with detailed models of the spinal cord, as already implemented for mouse models [71]. Furthermore, other aspects of human walking modulation involving higher voluntary control should be investigated. More experiments could target the modeling of specific motor behaviors, such as high ground clearance, stairs climbing, walking on slopes, obstacle avoidance. Moreover, the investigation of bioinspired controllers by including neural feedback and feedforward mechanisms would permit to explore other neuromotor control theories in human locomotion, such as the proximal-distal gradient control observed in animal experiments [72].

## Conclusion

In this work, we investigated the nature of sensory reflex modulation potentially behind the generation of different walking behaviors using neuromuscular simulation tools. We focused on studying the modulation of speed, step length, and step duration, identifying the main reflexes that could be responsible for the modulation of the three gait characteristics. Hamstrings length stretch reflexes and plantarflexors positive force feedbacks during stance were found to have a primary effect on step length modulation. On the other hand, stretch reflexes of iliopsoas, hamstrings, gluteus maximus, and tibialis anterior active during pre-swing, swing, and landing were found to modulate both step length and step duration. These reflexes were found to be sufficient and necessary to modulate a wide range of the three gait characteristics under analysis. Furthermore, the solutions obtained showed similarities with previous experimental studies on gait modulation in terms of kinematics, ground reaction forces and muscle activation [36], [46], [33], [54], [55] and with experiments investigating the activation of sensory afferents in human walking [56], [65], [68], [69]. This study provides a first contribution of the modulation of human locomotion in simulation environments based on physiologically relevant neural feedback circuits. Future directions should focus on investigating the joint contribution of feedforward and feedback neural components in the modulation of human gait as well as the potential neural mechanisms behind the sensory feedback modulation.
